# Emotion recognition deficits in eating disorders are explained by co-occurring alexithymia

**DOI:** 10.1098/rsos.140382

**Published:** 2015-01-21

**Authors:** Rebecca Brewer, Richard Cook, Valentina Cardi, Janet Treasure, Geoffrey Bird

**Affiliations:** 1 MRC Social, Genetic and Developmental Psychiatry Centre, Institute of Psychiatry, King's College London, London WC2R 2LS, UK; 2 Section of Eating Disorders, Psychological Medicine, Institute of Psychiatry, King's College London, London WC2R 2LS, UK; 3 Department of Psychology, City University London, London EC1R 0JD, UK; 4 Institute of Cognitive Neuroscience, University College London, London WC1N 3AR, UK

**Keywords:** alexithymia, facial expressions, emotions, face perception, eating disorders

## Abstract

Previous research has yielded inconsistent findings regarding the ability of individuals with eating disorders (EDs) to recognize facial emotion, making the clinical features of this population hard to determine. This study tested the hypothesis that where observed, emotion recognition deficits exhibited by patients with EDs are due to alexithymia, a co-occurring condition also associated with emotion recognition difficulties. Ability to recognize facial emotion was investigated in a sample of individuals with EDs and varying degrees of co-occurring alexithymia, and an alexithymia-matched control group. Alexithymia, but not ED symptomology, was predictive of individuals' emotion recognition ability, inferred from tolerance to high-frequency visual noise. This relationship was specific to emotion recognition, as neither alexithymia nor ED symptomology was associated with ability to recognize facial identity. These findings suggest that emotion recognition difficulties exhibited by patients with ED are attributable to alexithymia, and may not be a feature of EDs *per se*.

## 2. Introduction

Feeding and eating disorders (hereafter EDs) are axis I disorders characterized by disturbed and inappropriate patterns of eating [1]. Three subtypes are recognized, namely anorexia nervosa (AN; associated with emaciation, distorted body image and a fear of gaining weight), bulimia nervosa (BN; associated with periods of bingeing, followed by inappropriate compensatory behaviour) and more recently, binge eating disorder (BED; characterized by binge eating in the absence of the inappropriate compensatory behaviours associated with BN). Although not a diagnostic criterion, it is also widely believed that EDs are associated with atypical social and emotional functioning [2,3]. In particular, several authors have reported deficits of facial emotion recognition in individuals with AN and BN [4–7] and in non-clinical samples of women with high levels of ED symptomology [8,9]. This evidence is contested, however, with several studies finding no evidence for impaired recognition of facial emotion in ED samples [10–12]. Two recent meta-analyses of emotion recognition in ED [2,13] highlighted the discrepancies between studies, noting that where observed, group differences in the recognition of basic facial emotions are often small. In fact, the overall group difference fell short of significance in a meta-regression [13].

Achieving a better understanding of putative emotion recognition deficits in ED populations represents an urgent challenge to the field. Impaired recognition of others' emotions can impede the fluidity of social interactions and development and maintenance of relationships, by making it difficult for individuals to respond appropriately to others' expressions of emotion. Where observed in clinical populations, affective difficulties may strain patients' relationships, increasing the burden on themselves and their support networks. Problems interpreting emotion may also contribute to and exacerbate clinical symptoms, thereby impacting on treatment outcomes [14]. However, the current literature on emotion recognition in EDs, characterized by inconsistency, does not support reliable conclusions about the nature of EDs, and hampers the design and implementation of effective interventions. Beyond methodological differences [2], it is possible that differing levels of co-occurring alexithymia present in ED samples have contributed to this inconsistent literature.

Alexithymia is a non-clinical condition associated with problems identifying and describing one's own emotions [15]. Alexithymia frequently co-occurs with many developmental and psychological disorders, including autism spectrum disorder (ASD), panic disorder, somataform disorders and substance abuse [16]. Critically, however, alexithymia is an independent construct; many individuals experience alexithymia without receiving a clinical diagnosis, and the presence of alexithymia is not a necessary criterion for clinical diagnoses. Consistent with evidence that neurocognitive mechanisms responsible for the subjective experience of emotion make a causal contribution to the recognition of emotion in others, alexithymia is negatively associated with emotion recognition ability; difficulties recognizing emotions—of negative valence in particular—are well established in non-clinical alexithymic samples, and have recently been extended to clinical populations (see [16] for a review).

The alexithymia hypothesis of affective impairment [17] suggests that co-occurring alexithymia is responsible for equivocal reports of emotion recognition deficits across several clinical disorders. Studies where the incidence of alexithymia in clinical samples exceeds that present in control groups may be more likely to report emotion recognition deficits, than studies where levels of alexithymia are similar across groups. Despite the well-established association between alexithymia and emotion recognition abilities, few investigations of socio-emotional processing in clinical populations report alexithymia levels, and fewer still employ control groups matched for alexithymia, or control for co-occurring alexithymia statistically. Support for the alexithymia hypothesis is growing; in ASD samples, individual differences in facial emotion recognition [18], ability to interpret vocal [19] and musical [20] affect, and neural markers of empathy [21], are predicted by co-occurring alexithymia, and not ASD *per se*. Similarly, putative deficits of emotion recognition in individuals with somatoform disorder, relative to control participants, do not remain once alexithymia levels are controlled for [22].

Alexithymia co-occurs with all ED subtypes [23,24], and with non-clinical ED symptomology [8,25]. Despite high co-occurrence between EDs and alexithymia, however, these constructs are distinct [26]. Significantly, poor emotion recognition and high levels of alexithymia have previously been reported in the same ED sample [27], raising the possibility that alexithymia, not ED symptomology, is responsible for emotion recognition difficulties.

We tested the hypothesis that, where observed, difficulties recognizing facial emotion experienced by ED patients are not a symptom of EDs, but are instead owing to alexithymia. Individuals with EDs and an alexithymia-matched control group completed a novel emotion recognition procedure, whereby tolerance to visual noise indexed ability to recognize facial emotion. A comparable identity recognition procedure was employed as a control task. We hypothesized that alexithymia, but not ED symptomology, would predict facial emotion recognition abilities, and that these would not differ between clinical and alexithymia-matched control groups.

## 3. Method

### 3.1 Participants

Twenty-one females with an ED (19 AN, two BN; mean age =23.38 years, s.d. =6.31) and 21 females with no past or present psychological disorder (mean age =25.67 years, s.d. =6.57) participated in the study. Pilot data obtained using the current task in a group of 12 typical individuals were used in a power analysis, conducted using GPower, to indicate that the correlation between alexithymia and global emotion threshold (with effect size of 0.483) should reach significance (where power =0.95, *α*=0.05) with a sample size of 38. As pilot data did not include an ED group, however, and the current task differs from emotion recognition tasks previously used, a power calculation could not be performed in order to determine the necessary sample size for the group difference to reach significance. As previous ED emotion recognition studies have employed a minimum of 20 participants in each group [2], the sample size indicated by the power analysis was increased, to include 21 participants in both the ED and control groups.

All participants gave informed consent prior to participation. All individuals with an ED were diagnosed by independent clinicians, according to DSM-IV [28] or DSM-V [1] criteria. Fourteen of the 21 ED participants, diagnosed more than 1 year prior to study participation, also completed the EDs section of the research version of the structured clinical interview for DSM-IV (SCID) [29]. One member of the ED group had not previously been diagnosed by a clinician, but scored above the recommended cut-off for clinical significance on the eating disorder examination questionnaire (EDE-Q) [30], had a BMI below the healthy weight cut-off (16.5) and met criteria for ‘anorexia, binge-purging type’ according to the SCID. This participant was not an outlier in any analysis, and her exclusion did not alter the pattern of results. The ED group had significantly higher ED symptomology (*M*=4.19, s.d. =1.66), as assessed by the EDE-Q, than the control group (*M*=1.30, s.d. =1.36) [*t*_40_=6.169,*p*<0.001,*d*=1.951, CI(1.94,3.83)].

The ED and control groups were matched for age [*t*_40_=1.15,*p*=0.257,*d*=0.364, CI(−6.30,1.73)], IQ measured by the Wechsler abbreviated scale of intelligence [31] [*t*_40_=0.57,*p*=0.578,*d*=0.180, CI(−4.58,8.11)], and alexithymia measured by the Toronto alexithymia scale (TAS-20) [32] [*t*_40_=1.24,*p*=0.222,*d*=0.392, CI(−14.03,3.36)]. Seven participants (33%) in the control group (*M*=49.95, s.d. =15.45) and nine participants (42%) in the ED group (*M*=55.29, s.d. =12.25) met the criterion for severe alexithymia (TAS-20 score > 60). TAS-20 scores ranged from 26 to 79 in the control group, and 26 to 73 in the ED group. Although alexithymia and depression are independent constructs [33], the importance of controlling for depression in studies of EDs and alexithymia is well established [34,35]. Depression levels were therefore measured in all participants using the depression, anxiety and stress scale [36]. Depression was significantly associated with both ED symptomology (*r*_40_=0.655,*p*<0.001) and alexithymia (*r*_40_=0.384,*p*=0.012), and differed significantly between the ED (*M*=22.26, s.d.=11.08) and control groups (*M*=5.38, s.d.=6.98) [*t*_40_=5.91,*p*<0.001,*d*=1.869, CI(11.11, 22.66)]. All questionnaire measures were completed prior to the experiment.

### 3.2 Stimuli and materials

**Figure 1.**
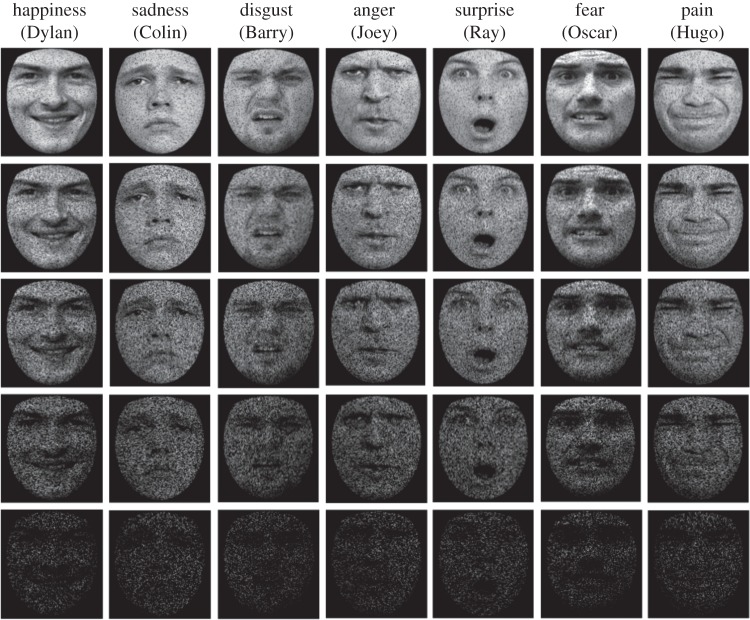
Examples of stimuli, showing all seven emotions (happiness, sadness, disgust, anger, surprise, fear and pain) each expressed on one of the seven identities (Dylan, Colin, Barry, Joey, Ray, Oscar and Hugo). During the experimental procedure, each emotion was equally likely to be expressed by each of the seven identities, ensuring that identity and emotion were not confounded. Examples show stimuli obscured by increasing levels of visual noise (10%, 30%, 50%, 70% and 90%).

Emotional face stimuli were taken from the STOIC database [37] and supplemented by stimuli purposely created for the study, yielding a total of 49 stimuli. The stimulus set used in the test phase comprised seven male identities, expressing happiness, sadness, surprise, fear, disgust, anger and pain (see figure 1 for examples). An additional seven images showing the seven identities with an emotionally neutral expression were used during training. Each stimulus was a greyscale image depicting a face, cropped to remove external features. Stimuli were presented on a 15^′′^ LCD screen and subtended approximately 8°×7° when viewed at a distance of 60 cm.

### 3.3 Training procedure

Two trial-to-criterion training phases, whereby participants were required to meet predetermined criteria before progressing, preceded the experimental task, allowing participants to learn the names of the seven identities. In the first training phase, participants viewed seven emotionally neutral identities and were prompted to select a particular identity (e.g. ‘pick Oscar’) using key presses. The location of each identity was randomized throughout. The second training phase ensured that participants could correctly identify each face when presented in isolation; i.e. without the other identities present for comparison. Participants viewed one facial identity and had to select the correct name from the seven options. A screen showing the seven identities with their corresponding names preceded both training tasks. Stimuli remained visible until participants responded, and were followed by accuracy feedback alongside the correct answer. On both training tasks, participants were required to correctly and consecutively identify the seven individuals twice in order to proceed. This ensured that participants had sufficiently memorized the identities. Participants did not receive training in emotion recognition, nor was their ability to recognize emotions measured prior to the test procedure, as this may have altered participants' recognition performance in the experimental paradigm, and emotional expressions are encountered in daily life, unlike the novel identities.

### 3.4 Test procedure

Experimental trials began with a fixation point (1000 ms) followed by a single facial image, depicting one of the seven possible identities exhibiting an emotional expression (800 ms). This was replaced by a prompt to attribute either emotion (e.g. ‘anger: yes or no?’) or identity (e.g. ‘Oscar: yes or no?’). Importantly, identical stimuli were used to test emotion and identity recognition, and emotion and identity trials were interleaved. Participants were therefore unaware whether they would have to judge identity or emotion when viewing each stimulus. This feature of the design ensured that both attributes had to be processed simultaneously, as is the case in real-life interactions. The attribution prompt remained visible until participants responded with a key press.

Participants' emotion and identity recognition ability was estimated by determining their tolerance to high-frequency visual noise (figure 1). An adaptive staircase procedure was used whereby the amount of noise superimposed on each stimulus image was varied incrementally, to determine the maximum level of noise participants could tolerate and still recognize each emotion and identity reliably (hereafter their recognition threshold). Higher thresholds are indicative of superior recognition ability. Initial threshold estimates for each of the seven identities and emotions were set at 50%, and remained at this level for the first 84 trials (six presentations per emotion and identity). Thereafter, if participants correctly recognized a particular emotion or identity twice consecutively, or made a single incorrect response, that noise parameter was increased (making that attribution harder on subsequent trials) or decreased (making that attribution easier on subsequent trials), respectively. The noise manipulation was achieved by replacing a given proportion (initially 50%) of the greyscale intensity values comprising the image with zeros, setting them to black. Intensity values were selected for distortion at random, sampling the entire facial image uniformly.

The size of each stepwise increment decreased as participants progressed through the experiment. From the 85th trial until the 140th trial, stepwise adjustments of ±16% were made. In the second, third, fourth and fifth blocks (each comprising 140 trials), the stepwise adjustments were decreased to ±8%, ±4%, ±2% and ±1%, respectively. Large increments early in the procedure ensured that the staircase quickly arrived at the approximate threshold for each identity and emotion. Smaller increments towards the end of the procedure ensured that threshold estimates became more stable as participants approached their maximum level of tolerance, and allowed estimates to be ‘fine-tuned’. The 14 threshold estimates (seven emotions, seven identities) reached after 700 trials (50 trials per emotion and identity) were taken as the final recognition thresholds for that participant. Prior to the experimental paradigm, participants completed five practice trials to familiarize themselves with the format of experimental trials.

A subset of individuals who took part in the pilot study (eight typical participants) completed the task a second time, in order for test–retest reliability of the current paradigm to be determined. Test–retest reliability analysis revealed a trend for global emotion thresholds to correlate across the two time points (*r*=0.513,*p*=0.073), and a significant correlation between global identity thresholds across the two time points (*r*=0.641, *p*=0.018). Importantly, this suggests that a specific deficit in emotion recognition could not simply be explained by reduced reliability of the identity task.

## 4. Results

In addition to the 14 recognition thresholds (seven emotions, seven identities) estimated for each participant, global emotion and identity thresholds were calculated by averaging across the seven individual emotion and identity estimates. These global measures of recognition ability for identity and emotion are directly comparable—each is a composite of the thresholds estimated for seven categories, comprising seven exemplars. Associations between the resulting distributions, alexithymia and ED symptomology were then determined (table 1).

**Table 1.** Means and standard deviations for recognition thresholds demonstrated by control and eating disorder (ED) groups, with *t*-tests for group differences, and correlations with alexithymia and ED symptomology. (Alexithymia is measured by the Toronto alexithymia questionnaire (TAS-20), whereas ED symptomology is measured by the eating disorder examination questionnaire (EDE-Q). None of the measures of emotion or identity recognition was associated with ED symptomology. Alexithymia was significantly negatively correlated with the threshold for global emotion recognition, and for happiness, disgust and pain recognition. There was also a strong trend for alexithymia to be negatively correlated with anger recognition threshold. *p<0.05; ^**^p<0.025.)

attribute	control mean (s.d.) %	ED mean (s.d.) %	group contrast	correlation with EDE-Q	correlation with TAS-20
emotion	62.0 (11.3)	57.3 (18.8)	*t*_40_=0.978	*r*=0.024	*r*=−0.340*
identity	47.3 (20.3)	43.8 (20.8)	*t*_40_=0.546	*r*=0.021	*r*=−0.226
happiness	70.9 (18.9)	76.1 (17.8)	*t*_40_=0.907	*r*=0.226	*r*=−0.305*
sadness	68.6 (14.3)	68.8 (26.3)	*t*_40_=0.029	*r*=0.151	*r*=−0.135
disgust	51.0 (19.6)	51.4 (24.5)	*t*_40_=0.056	*r*=−0.069	*r*=−0.381^**^
anger	53.8 (23.3)	45.2 (30.2)	*t*_40_=1.022	*r*=0.004	*r*=−0.301
surprise	84.2 (12.2)	73.6 (24.7)	*t*_40_=1.766	*r*=−0.055	*r*=−0.026
fear	38.5 (22.3)	34.0 (26.8)	*t*_40_=0.595	*r*=0.081	*r*=−0.133
pain	66.9 (21.9)	52.1 (27.0)	*t*_40_=1.947	*r*=−0.166	*r*=−0.323*

**Figure 2.**
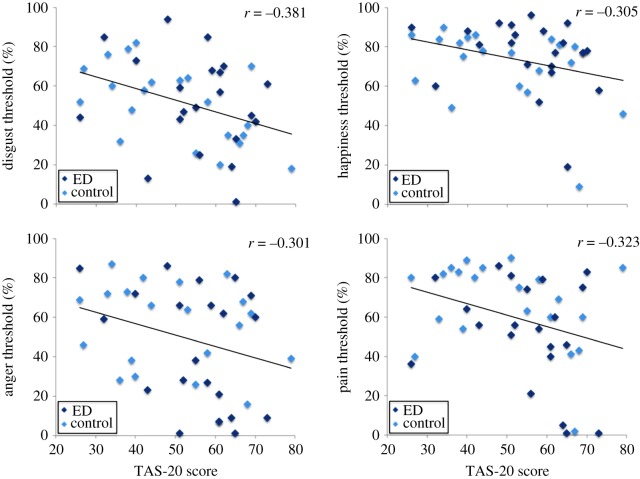
Scatter plots with best-fitting regression lines showing recognition thresholds for disgust, happiness, anger and pain recognition. Recognition thresholds are shown for individuals in the eating disorder and control groups, as a function of their score on the Toronto alexithymia scale (TAS-20). A greater score on the TAS-20 indicates more severe levels of alexithymia.

Alexithymia severity was negatively correlated with global emotion thresholds (*r*_40_=−0.340,*p*=0.027), but not with global identity thresholds (*r*_40_=−0.226,*p*=0.150). Significant simple correlations were observed between alexithymia severity and happiness threshold (*r*_40_=−0.305,*p*=0.049), disgust threshold (*r*_40_=−0.381,*p*=0.013) and pain threshold (*r*_40_=−0.323,*p*=0.037). The correlation between alexithymia and anger threshold also approached significance (*r*_40_=−0.301,*p*=0.053). Scatter plots depicting these simple correlations can be seen in figure 2. Recognition of the remaining emotions—sadness, surprise and fear—also showed negative correlations with alexithymia severity, but fell short of statistical significance.

In order to determine the impact of ED diagnosis on emotion and identity recognition, a 2 (ED group) × 2 (task: global emotion threshold versus global identity threshold), analysis of variance was performed on the emotion and identity recognition thresholds. Neither the main effect of ED group (*F*_1,40_=0.68,*p*=0.416,*η*=0.017), nor the ED group × task interaction (*F*_1,40_=0.05,*p*=0.819,*η*=0.001) was significant. Follow-up *t*-tests were conducted to separately assess the impact of ED group on each of the tasks individually. As predicted by the alexithymia hypothesis of affective impairment, individuals with EDs and control participants matched for co-occurring alexithymia demonstrated equivalent recognition thresholds for facial emotion (*t*_40_=0.978,*p*=0.334,*d*=0.309, CI(−0.05,0.14)) and identity (*t*_40_=0.546,*p*=0.588,*d*=0.137, CI(−0.09,0.16)). There was no difference between the groups in ability to recognize any of the seven individual emotions and no correlation between EDE-Q score and either emotion or identity recognition thresholds (table 1).

Together, these results suggest that ED symptomology is unrelated to emotion and identity recognition ability, whereas alexithymia explains substantial variation in participants' ability to recognize facial emotion. Nevertheless, it is important to determine whether the significant relationships observed between alexithymia and emotion recognition survive once individuals' depression scores are accounted for [34,35]. In addition, individual differences attributable to age and IQ—variables known to affect performance on face perception tasks—may prevent detection of simple correlations between ED symptomology and emotion recognition ability.

To address these issues, hierarchical regression analyses were conducted on the global emotion and global identity thresholds. Depression scores, age and IQ, were entered in the first step of the regression model, followed by alexithymia in the second step, and ED symptomology in the third. Alexithymia was found to be a significant predictor of the emotion recognition thresholds over and above the demographic variables (*t*_40_=2.422,*p*=0.020,*d*=0.766), and its addition to the model increased the variance accounted for by 10.1%. ED symptomology, when added in the third step of the model, did not predict emotion recognition threshold significantly and yielded a non-significant increase in the variance accounted for (2.3%). When global identity threshold was also controlled for in the first step of the model, the predictive ability of alexithymia fell to a two-tailed trend (*p*=0.065).

Although the control and ED groups were approximately matched for alexithymia, a significant correlation was observed between alexithymia and ED symptomology (*r*_40_=0.343,*p*=0.026). To ensure that multicollinearity between these variables did not obscure a relationship between ED symptomology and emotion recognition ability, a further regression analysis was conducted: depression scores, age and IQ were again entered in the first step, but now followed by ED symptomology in the second step, and alexithymia third. ED symptomology again failed to predict recognition thresholds for emotion, whereas alexithymia remained a significant predictor (*t*_40_=2.56,*p*=0.015,*d*=0.812), increasing the variance accounted for by 11.3%. See table 2 for a summary of the regression models.

**Table 2.** (*a*) Regression models for the prediction of emotion recognition threshold, including demographic variables (age, IQ and depression) in the first step, alexithymia, measured by the TAS-20 in the second step, and ED symptomology, measured by the EDE-Q in the third. (*b*) Regression models including demographic variables in the first step, ED symptomology in the second step, and alexithymia in the third. (Both hierarchical regressions indicate that alexithymia does, and ED symptomology does not, predict emotion recognition threshold over and above demographic variables, regardless of the order they were entered into the regression model.)

step	predictor	*β*	*p*	*R*^2^ (%)	Δ*R*^2^ (*p*)
(*a*)					
1	age	−0.012	0.001	25.9	25.9% (0.009)
	IQ	0.002	0.388		
	depression	−0.002	0.259		
2	age	−0.011	0.001	36.0	10.1% (0.020)
	IQ	0.002	0.257		
	depression	0.000	0.853		
	alexithymia	−0.004	0.020		
3	age	−0.011	0.001	38.4	2.3% (0.249)
	IQ	0.002	0.249		
	depression	−0.002	0.399		
	alexithymia	−0.004	0.015		
	ED symptomology	0.015	0.249		
(*b*)					
1	age	−0.012	0.001	25.9	25.9% (0.009)
	IQ	0.002	0.388		
	depression	−0.002	0.259		
2	age	−0.011	0.002	27.1	1.2% (0.439)
	IQ	0.002	0.389		
	depression	−0.003	0.178		
	ED symptomology	0.011	0.439		
3	age	0.011	0.001	38.4	11.3% (0.015)
	IQ	0.002	0.249		
	depression	−0.002	0.399		
	ED symptomology	0.015	0.249		
	alexithymia	−0.004	0.015		

In a convergent analysis, partial correlation coefficients were computed between emotion threshold and alexithymia, and emotion threshold and EDs, adjusted for age, IQ and depression. Partial coefficients were compared using Steiger's *Z*-test. Results showed a significantly stronger relationship between alexithymia and emotion threshold than that between emotion threshold and ED, whether ED was measured using EDEQ scores (*z*=1.75,*p*<0.05), or as a categorical variable (*z*=2.25,*p*=0.01).

Finally, hierarchical regression analyses were also conducted for the recognition thresholds calculated for the seven individual emotions. Recognition ability for happiness, anger and disgust was significantly predicted by alexithymia and not ED symptomology, having taken account of age, IQ and depression, irrespective of the order alexithymia and ED symptomology were entered into the model (table 3). Although a significant simple correlation was observed between alexithymia and recognition of pain, alexithymia failed to predict pain thresholds once the variance attributable to age, IQ and depression was accounted for (*p*=0.064). Overall, these results indicate that alexithymia, and not ED symptomology, explains emotion recognition.

**Table 3.** (*a*) Regression models for the prediction of happiness, anger, disgust and pain recognition thresholds, including demographic variables (age, IQ and depression) in the first step, alexithymia, as measured by the TAS-20 in the second step, and ED symptomology, as measured by the EDE-Q in the third. (For happiness, anger and disgust, alexithymia significantly predicts recognition threshold, above demographic variables, whilst ED symptomology is not predictive. For pain, once demographic variables are accounted for, the predictive ability of alexithymia falls short of significance.) (*b*) Regression models for the prediction of happiness, anger, disgust and pain recognition threshold, including demographic variables in the first step, ED symptomology in the second step, and alexithymia in the third. (As was the case when alexithymia was entered into the model before ED symptomology, for happiness, anger and disgust, it was alexithymia, and not ED symptomology, which significantly predicted recognition threshold, over and above demographic variables. For pain, alexithymia was no longer a significant predictor of recognition threshold once demographic variables were accounted for.)

step	predictor	*B*	*p*	*R*^2^	Δ*R*^2^ (*p*)	*β*	*p*	*R*^2^	Δ*R*^2^ (*p*)
(*a*)		happiness recognition threshold				anger recognition threshold			
1	age	−0.003	0.459	4.5%	4.5% (0.620)	−0.017	0.010	16.8%	16.8% (0.070)
	IQ	−0.002	0.400			0.002	0.561		
	depression	0.001	0.528			−0.001	0.840		
2	age	−0.003	0.479	17.6%	13.1% (0.020)	−0.016	0.008	27.4%	10.6% (0.025)
	IQ	−0.002	0.504			0.003	0.409		
	depression	0.004	0.127			0.002	0.487		
	alexithymia	−0.005	0.020			−0.007	0.025		
3	age	−0.003	0.518	23.8%	6.2% (0.097)	−0.016	0.009	27.5%	0.1% (0.866)
	IQ	−0.002	0.509			0.003	0.414		
	depression	0.001	0.797			0.002	0.654		
	alexithymia	−0.006	0.011			−0.007	0.027		
	ED symptomology	0.029	0.097			0.004	0.866		
		disgust recognition threshold				pain recognition threshold			
1	age	−0.013	0.014	15.4%	15.4% (0.092)	−0.010	0.101	18.2%	18.2% (0.052)
	IQ	0.001	0.829			0.007	0.081		
	depression	−0.002	0.456			−0.005	0.086		
2	age	−0.013	0.011	29.3%	13.9% (0.010)	−0.009	0.102	25.5%	7.3% (0.064)
	IQ	0.001	0.630			0.007	0.051		
	depression	0.001	0.772			−0.003	0.352		
	alexithymia	−0.006	0.010			−0.005	0.064		
3	age	−0.013	0.012	29.3%	0.0% (0.956)	−0.009	0.108	25.6%	0.0% (0.951)
	IQ	0.001	0.634			0.007	0.054		
	depression	0.001	0.845			−0.003	0.440		
	alexithymia	−0.006	0.012			−0.005	0.069		
	ED symptomology	0.001	0.956			0.001	0.951		
(*b*)		happiness recognition threshold				anger recognition threshold			
1	age	−0.003	0.459	4.5%	4.5% (0.630)	−0.017	0.010	16.8%	16.8% (0.070)
	IQ	−0.002	0.400			0.002	0.561		
	depression	0.001	0.528			−0.001	0.840		
2	age	−0.003	0.489	8.4%	3.9% (0.216)	−0.017	0.011	16.8%	0.0% (0.899)
	IQ	−0.002	0.397			0.002	0.566		
	depression	−0.001	0.740			0.000	0.946		
	alexithymia	0.023	0.216			−0.003	0.899		
3	age	−0.003	0.518	23.8%	15.3% (0.011)	−0.016	0.009	27.5%	10.7% (0.027)
	IQ	−0.002	0.509			0.003	0.414		
	depression	0.001	0.797			0.002	0.654		
	alexithymia	0.029	0.097			0.004	0.866		
	ED symptomology	−0.006	0.011			−0.007	0.027		
		disgust recognition threshold				pain recognition threshold			
1	age	−0.013	0.014	15.4%	15.4% (0.092)	−0.010	0.101	18.2%	18.2% (0.052)
	IQ	0.001	0.829			0.007	0.081		
	depression	−0.002	0.456			−0.005	0.086		
2	age	0.005	0.015	15.6%	0.2% (0.783)	−0.010	0.104	18.3%	0.1% (0.856)
	IQ	0.003	0.831			0.007	0.086		
	depression	0.004	0.703			−0.005	0.238		
	ED symptomology	0.021	0.783			−0.004	0.856		
3	age	0.005	0.012	290.3%	13.8% (0.012)	−0.009	0.108	25.6%	7.2% (0.069)
	IQ	0.003	0.634			0.007	0.054		
	depression	0.003	0.845			−0.003	0.440		
	ED symptomology	0.020	0.956			0.001	0.951		
	alexithymia	0.002	0.012			−0.005	0.069		

## 5. Discussion

According to the alexithymia hypothesis of affective impairment [17], where observed, emotion recognition deficits in individuals with EDs are, in fact, owing to co-occurring alexithymia and should not be regarded as a core feature of these conditions. To test this assertion, this study compared facial emotion recognition ability in individuals with an ED and an alexithymia-matched control group. Consistent with the alexithymia hypothesis of impaired emotion recognition, the ED and alexithymia-matched control groups demonstrated comparable recognition of facial emotion and identity. Crucially, however, alexithymia, not ED symptomology, was found to predict emotion recognition ability; severe alexithymia was associated with impaired emotion recognition, whereas ED diagnosis was unrelated to emotion recognition thresholds. Importantly, this relationship remained once age, IQ and depression, were taken into account.

These results shed light on the inconsistent literature on emotion recognition in ED populations. Several authors have reported evidence for impaired recognition of facial emotion in EDs [4–6], prompting speculation that atypical emotion processing may be an important feature of these conditions [2]. The current findings suggest that emotion recognition impairment is, in fact, unrelated to EDs *per se*, and that heterogeneity of ED samples, with respect to alexithymia, is probably responsible for many of the contradictory findings reported previously. Where impaired emotion recognition in ED samples has been reported, clinical groups may have contained a greater proportion of individuals with severe alexithymia than control groups [27].

The current findings further support the suggestion that co-occurring alexithymia may explain inconsistent reports of impaired emotion recognition across a range of disorders [17]. Many conditions are associated with elevated rates of alexithymia, and equivocal reports of emotion recognition deficits [16]. Recognition of facial emotion in autism was predicted by levels of co-occurring alexithymia and not by the presence or severity of autism [18]; a pattern replicated in this study with an ED sample. These convergent findings suggest that co-occurring alexithymia can produce similar emotion recognition difficulties in different clinical conditions. It is therefore crucial that future studies of emotion recognition in clinical populations match control groups for alexithymia, or control for its influence statistically, allowing researchers to test whether condition symptomology makes an independent contribution to emotion recognition.

The individual emotion thresholds most strongly related to alexithymia in this study were happiness, disgust and anger. While the relationship with disgust and anger recognition accords well with Cook *et al*.'s [18] recent findings in ASD, the association with happiness recognition is observed less often, and contradicts the view that alexithymia is disproportionately related to impaired recognition of emotions with negative valence (see [16] for a review). Where recognition deficits are restricted to negative emotions, it is unclear whether this pattern reflects the ease with which happiness may be discriminated. Happiness is often the only emotion studied with a positive valance and happy expressions have highly distinctive local features [38]. In this study, ceiling effects were avoided by increasing difficulty for each emotion independently, by altering levels of visual noise based on performance. In addition, although the current procedure ensured that pain and happiness were assessed independently, the presence of pain is likely to have made happiness harder to discriminate than in previous studies, owing to expressions of pain sharing more physical features with happiness expressions than do other negative facial emotions.

The influence of co-occurring alexithymia may extend beyond expression recognition, potentially explaining a wide range of emotion processing difficulties observed in EDs. Alexithymia is associated with impaired performance when judging protagonists' emotions from vignettes, in ED participants [39] and individuals with non-clinical disordered eating [8]. While the independent contributions of ED and alexithymia were not addressed, these findings suggest that the impact of co-occurring alexithymia in ED samples may extend to broader socio-emotional abilities. We also note that co-occurring alexithymia is known to be responsible for the difficulties interpreting vocal [19] and musical affect [20], and the reduced empathy [21], seen in some individuals with ASD.

The current ED sample did not contain sufficient numbers of BN or BED patients to determine how the association with alexithymia varies as a function of ED subtype. Alexithymia co-occurs with all subtypes [23,24], however, and neither BN patient was an outlier. Determining whether alexithymia produces equivalent deficits of facial emotion recognition in each subtype remains a priority for future research, especially as only one study has directly compared emotion recognition ability across ED subtypes [23]. Similarly, while the sample size was modest, it was sufficient to observe reliable associations between alexithymia and emotion recognition ability. A power calculation suggested that over 4500 patients would be required for the observed effect of EDs on emotion recognition to reach significance, suggesting the effect of alexithymia on emotion recognition is an order of magnitude greater than that of EDs.

As expected, no association was seen between alexithymia or ED symptomology and identity recognition ability. Although a weak negative relationship was observed between alexithymia and identity recognition, this may reflect top-down effects, whereby individuals with high alexithymia, aware of their emotion recognition problems, attend to cues to facial emotion at the expense of identity recognition. Indeed, anecdotal accounts during debriefing suggest this may be the case. Short presentation durations coupled with the demand to process identity and emotion simultaneously, may have proved challenging for individuals with high levels of alexithymia. That no relationship was seen between alexithymia and identity recognition thresholds confirms that alexithymia is associated with problems of emotion interpretation and not simply impaired interpretation of degraded visual images.

Overall, these findings suggest that levels of co-occurring alexithymia, and not ED symptomology, predict ability to recognize facial emotion, in individuals with and without EDs. These results have significant implications for the conceptualization of EDs—suggesting that disordered emotion processing may not be a core feature of these conditions—as well as for socio-emotional research practice—highlighting the need to measure and control for the influence of co-occurring alexithymia when testing clinical populations. Together with previous findings, these results suggest that alexithymia may explain individual differences in affective processing across a range of clinical conditions.

## Supplementary Material

Data file - ‘Data Brewer Cook Cardi Treasure Bird’

## Data accessibility

The dataset supporting this article is available as the electronic supplementary material.
